# The safety and efficacy of N-acetylcysteine as an augmentation in the treatment of obsessive-compulsive disorder in adults: a systematic review and meta-analysis of randomized clinical trials

**DOI:** 10.3389/fpsyt.2024.1421150

**Published:** 2024-09-23

**Authors:** Shayan Eghdami, Negin Eissazade, Mohsen Heidari Mokarar, Mahsa Boroon, Laura Orsolini, Mohammadreza Shalbafan

**Affiliations:** ^1^ Brain and Cognition Clinic, Institute for Cognitive Sciences Studies, Tehran, Iran; ^2^ School of Medicine, Iran University of Medical Sciences, Tehran, Iran; ^3^ Department of Psychiatry, Imam Hossein Hospital, School of Medicine, Alborz University of Medical Sciences, Karaj, Iran; ^4^ Unit of Clinical Psychiatry, Department of Neurosciences/DIMSC, Polytechnic University of Marche, Ancona, Italy; ^5^ Mental Health Research Center, Psychosocial Health Research Institute (PHRI), Department of Psychiatry, School of Medicine, Iran University of Medical Sciences, Tehran, Iran

**Keywords:** acetylcysteine, obsessive-compulsive disorder, glutamate, systematic review, meta-analysis

## Abstract

**Background:**

Obsessive-compulsive disorder (OCD) ranks as the fourth most prevalent psychiatric disorder, with selective serotonin reuptake inhibitors (SSRIs) as its mainstay pharmacological treatment. However, approximately 40 to 60% of patients do not adequately respond to initial treatment, highlighting the need for alternative options. N-acetylcysteine (NAC) is one of the several medications that have been used in augmentation with SSRIs to enhance their efficacy.

**Objectives:**

We aimed to investigate the safety and efficacy of NAC, a glutamate-modulating agent, as an augmentation in the treatment of moderate to severe OCD.

**Method:**

We conducted a thorough search across PubMed, Scopus, Web of science, and ProQuest to identify relevant trials published until December 2023. The primary outcome of interest was the mean difference between the Yale-Brown Obsessive-Compulsive Scale (Y-BOCS) scores before and after administrating augmented NAC among patients with moderate to severe OCD. Furthermore, we compared the occurrence of adverse drug events between the experimental and control groups.

**Results:**

We included six randomized controlled trials with 195 patients. The results of our study indicated a positive outcome for the experimental group in terms of the total Y-BOCS score when using the medication for a period of five to eight weeks (p-Value = 0.05). However, no significant difference was observed for durations shorter than five weeks or longer than 12 weeks. Additionally, no significant difference was found between the two groups in terms of the obsession and compulsion Y-BOCS scores. Furthermore, no significant differences were observed in terms of adverse events.

**Conclusion:**

Augmentation of NAC with SSRIs may benefit patients with moderate to severe OCD. However, it is necessary to conduct additional multi-center trials over extended periods to develop a comprehensive strategy for action.

**Systematic review registration:**

https://www.crd.york.ac.uk/prospero/, identifier CRD42023463683.

## Introduction

Obsessive-compulsive disorder (OCD) is a complex and chronic psychiatric disorder affecting millions of individuals worldwide (1). It is characterized by obsessions, compulsions, or both, which not only consume a substantial amount of time but also impair daily functioning, often leading to considerable distress and a diminished quality of life. Obsessions are defined as persistent, intrusive thoughts, urges, or images that the individuals find difficult to dismiss or control. These obsessions often revolve around specific themes, such as fears of contamination, losing control, inappropriate sexual thoughts, religious concerns, or an overwhelming need for symmetry and perfection. In response, individuals with OCD engage in compulsions, repetitive behaviors or mental acts performed with the aim of reducing the anxiety associated with their obsessions. Common compulsions include excessive washing, checking, organizing, or performing mental rituals such as counting or repeating phrases (2).

The underlying pathophysiology of OCD involves a complex interplay of genetic, neurobiological, and environmental factors. Research has long suggested that various neurotransmitters, including serotonin, norepinephrine, acetylcholine, and dopamine, play crucial roles in the development and maintenance of OCD symptoms. This understanding has paved the way for the development of targeted pharmacological treatments (3). First-line treatments for OCD typically include selective serotonin reuptake inhibitors (SSRIs) and clomipramine. Additionally, cognitive behavioral therapy (CBT), particularly exposure and response prevention (ERP), is widely recognized as an effective non-pharmacological treatment for OCD. Despite the availability of these treatments, a significant proportion of patients, ranging from 40 to 60% (4–6), do not respond adequately to first-line therapies or experience intolerable side effects. This situation necessitates the search for efficacious alternative or adjunctive treatment options (7–10).

In recent years, increasing attention has been directed toward the glutamatergic system as a potential therapeutic target in OCD. Glutamatergic medications can act as receptor antagonists, reuptake inhibitors, co-agonists, and ion channel modulators. Altered glutamate levels, may contribute to the development and persistence of OCD symptoms (11). Glutamate has a significant role in the normal development of the cortico-striatal-thalamo-cortical (CSTC) circuitry, neural communication, plasticity, and overall brain function (12). For instance, elevated levels of glutamate and its byproducts are found in the basal ganglia, while reduced levels have been noted in the anterior cingulate cortex in patients with OCD (13). Additionally, disruptions in the balance of glutamate and its interaction with other neurotransmitters, such as GABA and serotonin, which are involved in the flow of glutamate between the cortex, thalamus, and basolateral amygdala, may result in abnormal CSTC pathway activity (14). Over the years, various glutamatergic agents have been investigated, such as lamotrigine, topiramate, memantine, glycine, D-cycloserine, riluzole and N-acetylcysteine (NAC) (15).

NAC, which has emerged as a particularly intriguing candidate, is a derivative of the amino acid cysteine, and has powerful antioxidant properties (16). It is well-known for its role in regulating intracellular levels of glutathione and modulating the glutamatergic system (17). Several clinical trials and studies have evaluated the efficacy of NAC as an adjunctive treatment in OCD, with some suggesting that it may offer significant benefits, particularly in patients who are resistant to conventional therapies. A systematic review and meta-analysis conducted in 2020 reported that NAC is a promising agent for the treatment of patient with OCD (18).

Given the evolving body of evidence regarding the use of NAC in the treatment of moderate to severe OCD, this systematic review and meta-analysis aims to comprehensively evaluate the literature, focusing on the safety and efficacy of NAC as an augmentation in the management of moderate to severe OCD.

## Methods

We followed the Preferred Reporting Items For Systematic Reviews And Meta-Analyses (PRISMA) reporting guidelines and the Cochrane Collaboration Handbook (19, 20) (PROSPERO ID: CRD42023463683).

### Eligibility criteria

The inclusion criteria for the studies were determined using the PICOT (population, intervention, comparison, outcome, time) framework. Studies were included if they involved individuals over the age of 18 who were diagnosed with OCD, according to either fourth or fifth edition of the Diagnostic and Statistical Manual of Mental Disorders (DSM-IV or DSM-5), and had a total Yale-Brown Obsessive-Compulsive Scale (Y-BOCS) score greater than 16, which is indicative of moderate to severe OCD (21). The intervention of interest was the use of NAC as an augmentation therapy for OCD. The comparison group consisted of patients receiving treatment with SRIs and SSRIs. The primary outcome was the reduction in OCD symptom severity, as measured by the Y-BOCS, a 10-item scale with total scores ranging from 0 to 40, where higher scores indicate more severe symptoms. Included studies were required to have a trial duration of at least 3 weeks to ensure sufficient time for assessing the effects of NAC on OCD symptoms. Quasi-experimental studies, observational studies, case series, and case reports, as well as conference abstracts, protocols, opinion pieces, theses, and review articles were excluded.

### Databases and search procedure

We conducted the search across electronic databases of PubMed, Web of Science, Scopus (as databases of journal article) and ProQuest (as a database of grey literature) to identify relevant trials, using the search strategies outlined in *Appendix 1* to collect the available evidence from original articles published until December 2023 in English. The search results were imported into an EndNote library (Version 20, Thomson Reuters, USA), and duplicated citations were eliminated using the “Find Duplicates” feature of the software.

### Outcome measures

The main measure of interest for our study was the effect size of Y-BOCS scores, which was determined by calculating the mean difference of scores before and after administrating NAC in augmentation with an SSRI. The secondary measure was the effect size of adverse events of NAC throughout the trials.

### Study selection

To ensure the accuracy and relevance of the study, two authors (SE and NE) independently screened the titles and abstracts of the selected studies. Following this, the full texts of the remaining articles were obtained and examined, according to the specified inclusion criteria. In the event of any disagreements, the authors resolved them through discussion or sought consultation with a third author (MSH).

In order to identify additional relevant papers, the references cited in the selected articles were also evaluated. The full texts of the studies that were ultimately selected underwent a comprehensive evaluation for quality assessment, data extraction, and analysis.

### Data extraction and quality assessment

Detailed data extraction was performed based on the pre-designed data extraction forms, including the study design, first author’s name, country, participants characteristics, interventions and outcomes.

Two authors (SE and NE) independently assessed the risk of bias in studies across six domains: random sequence generation (selection bias for controlled trials), allocation concealment (selection bias for controlled trials), blinding of participants and personnel (performance bias), blinding of outcome assessment (detection bias), incomplete outcome data (attrition bias), and selective reporting (reporting bias) (22).

### Evidence synthesis

We adopted systematic methods, including textual descriptions and tabulation, using the Review Manager (RevMan) (Version 5.4. the Cochrane collaboration, 2020), for evidence synthesis. We employed statistical techniques to estimate the missing values using the information provided in the articles. Specifically, we used Wan’s method to calculate the mean and standard deviation based on the sample size, median, range, and interquartile range (23). In cases where data were missing, we considered the participants as non-abstainers. We conducted a meta-analysis using RevMan to determine the weighted average treatment effect across the included studies. To assess heterogeneity among the studies, we utilized the chi-square statistic and calculated I^2^. Where I^2^ values greater than 40% or a chi-square statistic with a p-value less than 0.1 indicated significant statistical heterogeneity, a random-effects model was employed (24). To assess the effectiveness of the pharmacological intervention, we calculated the mean difference (MD) of total, compulsion and obsession Y-BOCS scores before and after the intervention. Additionally, we performed a subgroup analysis based on the period of intervention to address any clinical heterogeneity. We used relative risk (RR) with a 95% confidence interval (CI), to assess the occurrence of adverse events (25). To ensure consistency, we utilized data from the study that reported the longest follow-up period for each outcome.

## Results

### Description of included studies

**Figure 1:**
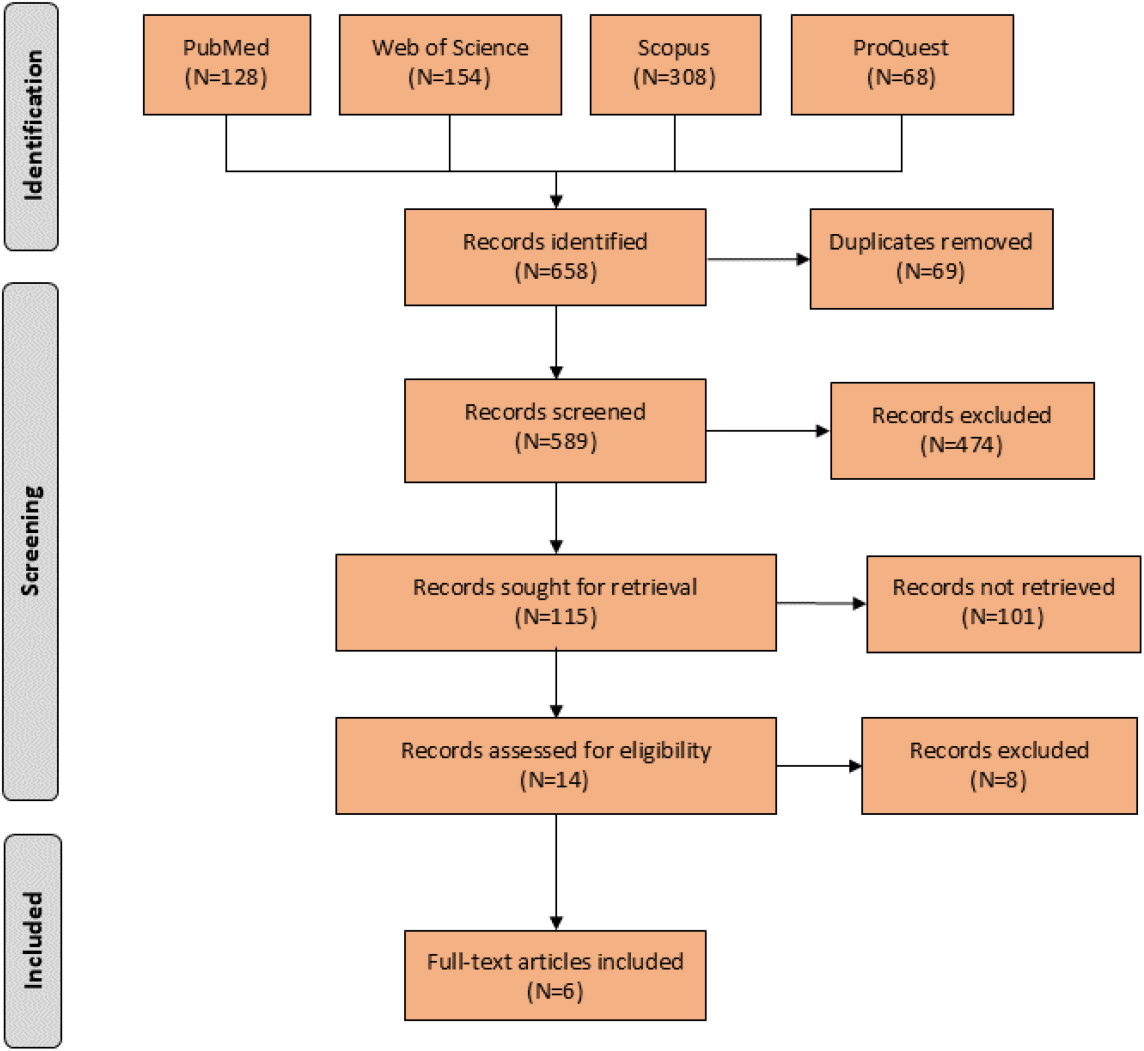
Flow diagram of the included studies.

**Figure 2:**
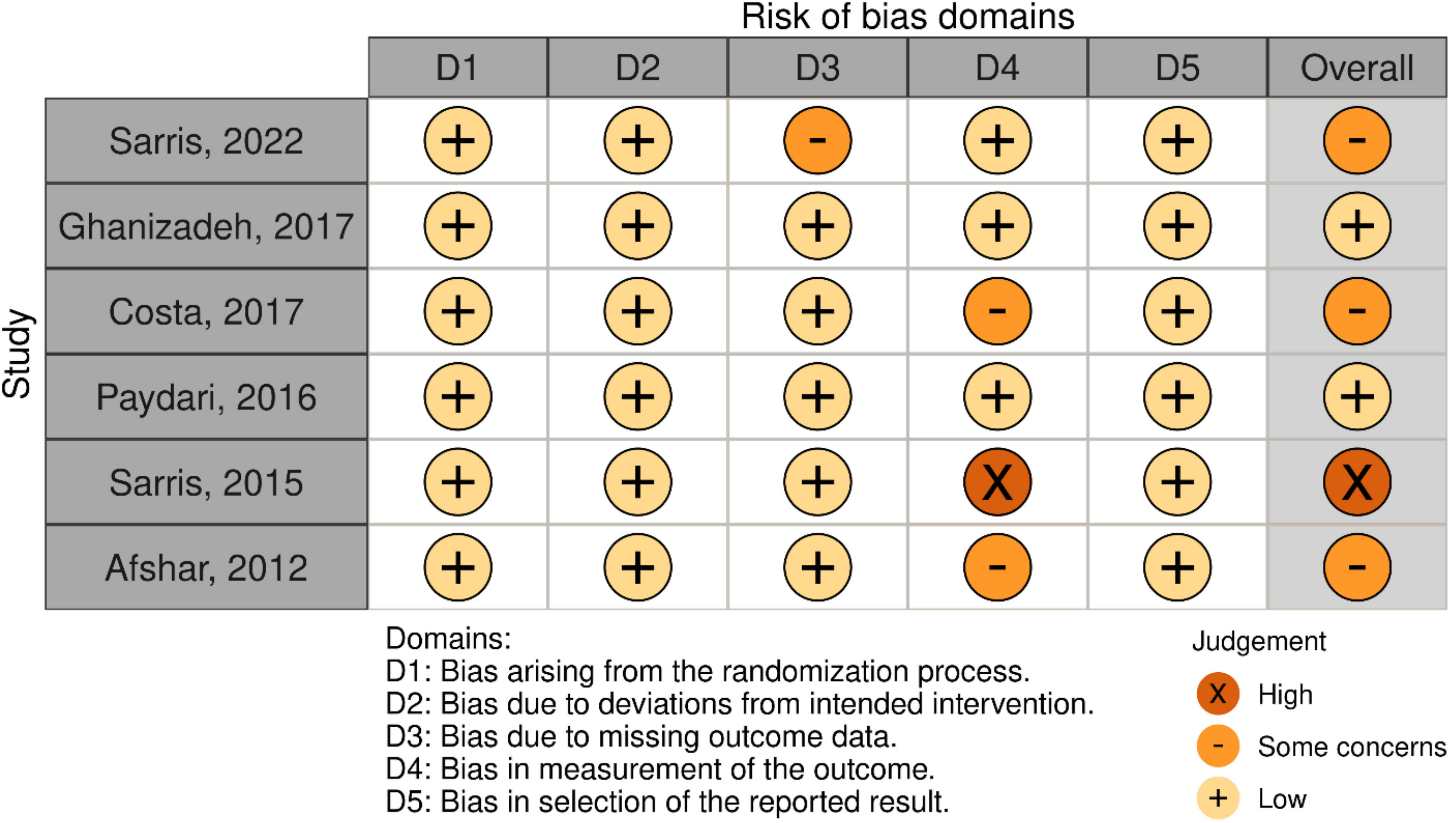
Risk of bias assessment.

Initial search resulted in 658 records. After assessing the titles and abstracts, a total of 14 studies were selected for full text screening, and six RCTs (26–31) with 195 participants with moderate to severe OCD met the inclusion criteria and were included in the meta-analysis (**Figure 1**). Their overall quality, as shown in **Figure 2**, was considered fair. The mean study duration was 15 weeks (ranging from 10 to 20). The dose of NAC ranged from 600 to 3000 mg/day. **Table 1** presents the summary of the included studies.

**Table 1:** Summary of clinical trials investigating the effect of NAC in treating moderate to severe OCD included in meta-analysis.

Study (first author, year of study)	Country	Participants		Follow-up time	NAC dose (initial dose-maximum dose) (mg/day)	Mean differences of Y-BOCS total score (endpoint -baseline), 95% CI, experimental group vs. control group	Mean differences of Y-BOCS Obsession score (endpoint -baseline), 95% CI, experimental group vs. control group	Mean differences of Y-BOCS compulsion score (endpoint -baseline), 95% CI, experimental group vs. control group
		Experimental group	Placebo group					
Sarris, 2022 (26)	Australia	29 Median age= 31.5 (20.8)	29 Median age=32.0 (21.0)	20 weeks	2000-4000	0.80 [-2.87, 4.47]	-0.28 [-2.29, 1.73]	0.92 [-1.06, 2.90]
Ghanizadeh, 2017 (27)	Iran	18 Mean age= 16.5 ± 2.9	11 Mean age= 15.9 ± 3.7	10 weeks	600-2400	-6.80 [-13.29, -0.31]	-1.00 [-2.63, 0.63]	-1.40 [-2.99, 0.19]
Costa, 2017 (28)	Brazil	17 Mean ag=37.8 ± 10.5	22 Mean ag=38.2 ± 11.3	16 weeks	1200-3000	-1.30 [-6.49, 3.89]	-0.30 [-1.95, 1.35]	-1.10 [-4.20, 2.00]
Paydari, 2016 (29)	Iran	22 Mean age=33.72 ± 11.25	22 Mean age=33.04 ± 11.44	10 weeks	1000-2000	-2.4 [-5.33, 0.53]	N/A	N/A
Sarris, 2015 (30, 32)	Australia	22 Mean age=39.14 ± 12.8	22 Mean age=34.86 ± 11.4	16 weeks	1000-3000	-0.28 [-6.29, 5.73]	-0.68 [-3.86, 2.50]	0.41 [-2.76, 3.58]
Afshar, 2012 (31)	Iran	19 Mean age=30.62 ± 5.35	20 Mean age=31.25 ± 4.70	12 weeks	600-2400	-5.14 [-7.05, -3.23]	N/A	N/A

### Total Y-BOCS scores

**Figure 3:**
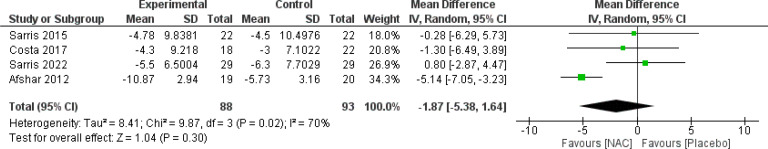
Comparison of total Y-BOCS scores between the NAC and control groups receiving NAC for 12 weeks and more. Also, the meta-analysis of two studies (27, 31) revealed that using NAC for five to eight weeks is significantly effective (MD = -2.95, 95% CI: [-5.87, -0.04], p-Value = 0.05, I^2^ = 0%) (**Figure 4**), while not significantly effective for four weeks or less (MD = 0.74, 95% CI: [-2.27, 3.74], p-Value = 0.63, I^2^ = 0%) (**Figure 5**).

**Figure 4:**
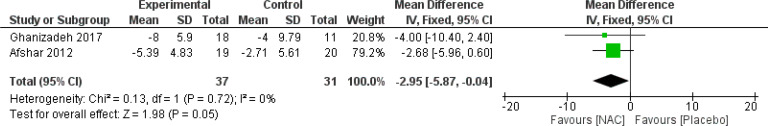
Comparison of total Y-BOCS scores between the NAC and control groups receiving NAC for five to eight weeks.

**Figure 5:**
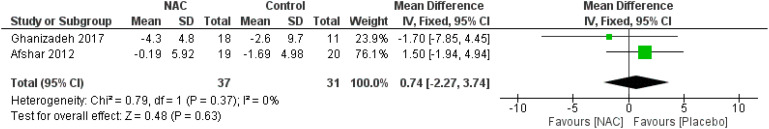
Comparison of total Y-BOCS scores between the NAC and control groups receiving NAC for four weeks and less.

The results indicated a trend toward the beneficial impact of NAC on total Y-BOCS scores over a duration of 12 weeks or longer (MD = -1.87, 95% CI: [-5.39, -1.66], p-Value = 0.30, I^2^ = 70%) (**Figure 3**) (26, 28, 30, 31). Also, the meta-analysis of two studies (27, 31) revealed that using NAC for five to eight weeks is significantly effective (MD = -2.95, 95% CI: [-5.87, -0.04], p-Value = 0.05, I2 = 0%) (**Figure 4**), while not significantly effective for four weeks or less (MD = 0.74, 95% CI: [-2.27, 3.74], p-Value = 0.63, I2 = 0%) (**Figure 5**).

### Obsession and compulsion Y-BOCS scores

**Figure 6:**
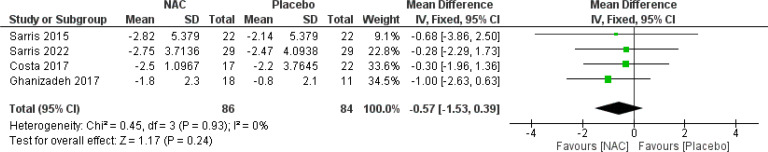
Comparison of obsession Y-BOCS scores between the NAC and control groups.

**Figure 7:**
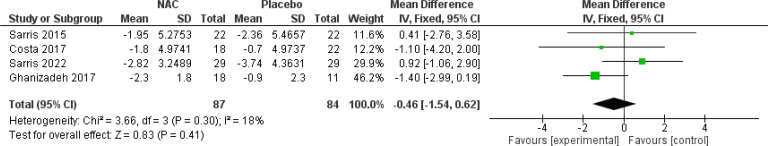
Comparison of compulsion Y-BOCS scores between the NAC and control groups.

The subgroup analysis of four studies (26–28, 30) revealed that NAC was not significantly effective for obsession (MD = -0.57, 95% CI: [-1.53, 0.39], p-Value = 0.24, I^2^ = 0%) or compulsion (MD = -0.40, 95% CI: [-1.65, 0.84], p-Value = 0.53, I^2^ = 18%) (**Figures 6**, **7**).

### Adverse events

**Figure 8:**
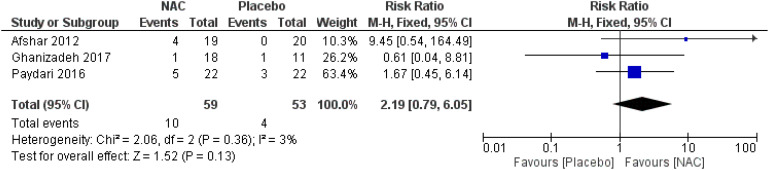
Comparison of the occurrence of diarrhea between the NAC and the control groups.

**Figure 9:**
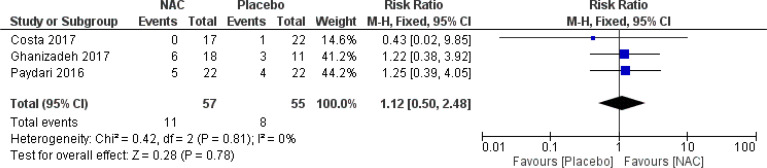
Comparison of the occurrence of drowsiness between the NAC and the control groups.

**Figure 10:**
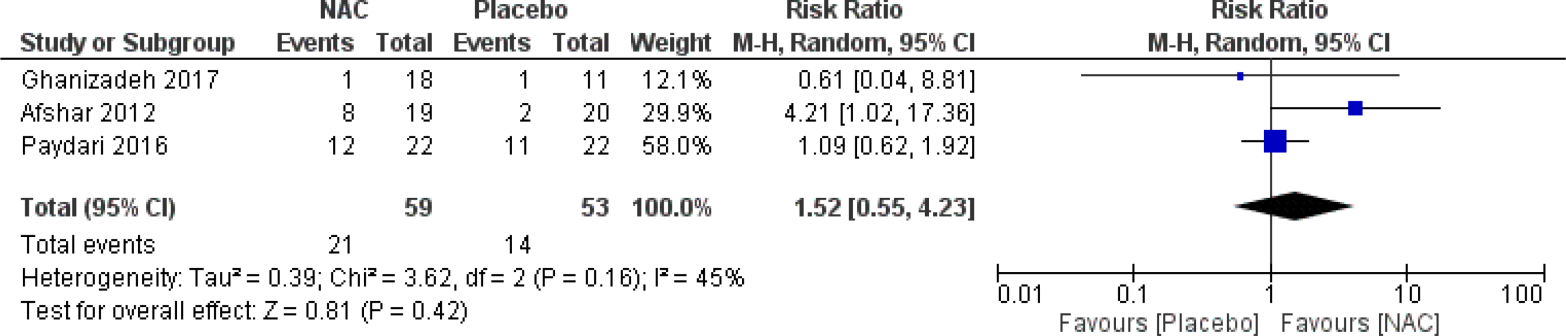
Comparison of the occurrence of nausea/vomiting between the NAC and the control groups.

**Figure 11:**
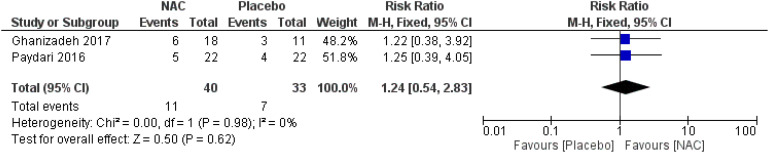
Comparison of the occurrence of dizziness between the NAC and the control groups.

**Figure 12:**
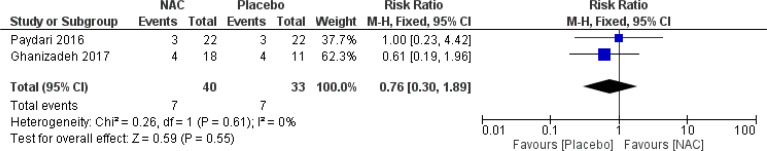
Comparison of the occurrence of dry mouth between the NAC and the control groups.

No significant differences were found on the occurrence of diarrhea, nausea/vomiting, dizziness, drowsiness, and dry mouth between the groups (**Figures 8**–**12**) (27–29, 31).

### Other outcomes

Ghanizadeh et al. (27) have reported that NAC had a positive impact on physical (p-Value = 0.005), emotional (p-Value = 0.001) and social (p-Value = 0.001) function after 10 weeks.

Afshar et al. (31) have reported that the severity of disorder, was significantly different between the NAC and control groups at week 12 (p-Value = 0.01). However, no significant difference was found for clinical improvement.

Costa et al. (28) have reported that there were no significant between-group differences in Dimensional Y-BOCS (DY-BOCS) (aggression/violence, sexual/religious, ordering/symmetry/counting, contamination/cleaning, hoarding, miscellaneous, and total), Beck Anxiety Inventory (BAI), Beck Depression Inventory (BDI), and Brown Assessment of Beliefs Scale (BABS) (insight/delusionality) scores.

## Discussion

We conducted a systematic review and meta-analysis on six RCTs to evaluate the safety and efficacy of NAC in the treatment of moderate to severe OCD. Our analysis revealed that NAC has a significant beneficial effect in managing moderate to severe OCD symptoms, between weeks five and eight. Notably, it was well-tolerated and causes only mild side effects, contributing to a favorable safety profile. The effect size, though favorable, was modest, raising questions about whether the observed changes would be perceived as meaningful in a clinical setting. The potential for greater clinical impact might emerge with longer study durations or higher doses of NAC. Indeed, while the studies included in our meta-analysis were limited in scope, there was a trend toward greater efficacy with increased doses of NAC. However, due to the small sample size and variability in study designs, this trend did not reach statistical significance for a period of longer than 12 weeks.

Moreover, our findings align with those of a 2020 systematic review and meta-analysis (18) of five RCTs (20, 21, 23, 26, 27), with a mean duration of 12 weeks (ranged from 10 to 16 weeks), and a maximum dose of NAC ranged from 2000 to 3000 mg/day. They have reported that NAC was statistically superior to placebo in reducing the Y-BOCS scores. Also, it was well-tolerated, with only mild side effects reported. However, our review suggests that the effect might be more nuanced, potentially influenced by factors such as dose and duration of treatment.

In the context of understanding the potential role of NAC in treating moderate to severe OCD, dysregulation of the CSTC circuit, specifically involving disruption of glutamatergic transmission, has been consistently observed in patients with OCD (33, 34). Notably, some studies have reported significantly elevated glutamate levels in the cerebrospinal fluid of patients with OCD, leading to excitotoxicity and oxidative stress, both of which are associated with the severity of OCD symptoms (11). Furthermore, drug-naive adults with OCD have exhibited higher levels of glutamate and glycine in their cerebrospinal fluid (35). Magnetic resonance spectroscopy has further revealed abnormalities in the glutamate-glutamine pathway (36). Moreover, preclinical studies suggest that NAC may influence oxidative stress (37), apoptosis (38), neurogenesis, neuroinflammation, mitochondrial dysfunction (39), and disruptions in the function of dopamine (40) and glutamate, as NAC transforms into cysteine in the CNS, which helps regulate the transportation of glutamate between nerve cells (11), through the glutamate/cystine antiporter (41), mainly found in glial cells (41). These findings collectively suggest that targeting glutamate activity with glutamatergic agents holds promise as a therapeutic strategy for individuals with OCD.

### Limitations

It is essential to acknowledge the limitations of this study. First, the inclusion criteria were restricted to English-language publications, potentially introducing language bias. Second, only six RCTs met our inclusion criteria. Third, the diversity in dosing regimens and follow-up periods across studies precluded subgroup analyses based on medication type and dosage. Fourth, the heterogeneity of the included studies in terms of patient populations, comorbidities, and study designs may have influenced the generalizability of the findings.

Future research should aim to conduct larger multi-center trials with longer follow-up periods. Moreover, investigations into the use of NAC in patients with comorbid disorders should be evaluated. Additionally, the impact of NAC on cognitive function (e.g., impairments in set-shifting ability, response inhibition, and nonverbal memory) (42) should be further investigated. Moreover, studies examining the potential benefits of NAC in combination with other emerging treatments, could provide valuable insights into comprehensive treatment strategies for OCD. Finally, a better understanding of patient-specific factors, such as genetic predispositions or comorbidities, may help identify those who are most likely to benefit from NAC therapy.

## Conclusions

Our systematic review and meta-analysis suggests that NAC may serve as an effective adjunct treatment for adults with moderate to severe OCD. Additionally, NAC was well-tolerated, with no serious adverse events reported. However, the potential for NAC to maintain or even enhance its therapeutic effects with prolonged use remains unclear, necessitating further high-quality large-scale RCTs for elucidating the optimal dosage and duration of NAC treatment, as well as exploring its mechanisms of action within the glutamatergic system.

## Data Availability

The original contributions presented in the study are included in the article/**Supplementary Material**. Further inquiries can be directed to the corresponding author.
