# Supplementary material for: The safety and efficacy of N-acetylcysteine as an augmentation in the treatment of obsessive-compulsive disorder in adults: a systematic review and meta-analysis of randomized clinical trials
Source: Front Psychiatry. 2024 Sep 23;15:1421150. doi: 10.3389/fpsyt.2024.1421150 (PMC11456833; doi:10.3389/fpsyt.2024.1421150)
Supplement: Supplementary file 1 [file DataSheet1.pdf]

Search strategy (Online Resource 1)

Databases

JOURNAL ARTICLES

1. Web of science
2. Medline
3. Scopus
4. CENTRAL
5. Proquest

Keywords:

1. randomized [Title/Abstract]
2. placebo [Title/Abstract]
3. randomly [Title/Abstract]
4. trial [Title/abstract]
5. randomized controlled trial [pt]
6. controlled clinical trial [pt]
7. clinical trial [pt]
8. #1 OR #2 OR #3 OR #4 OR #5 OR #6 OR #7

9. NAC [Mesh Terms]
10. Glutamate [Title/abstract]
11. Glutamatergic [Title/abstract]
12. Glutamic acid [Title/abstract]
13. acetylcysteine [Title/abstract]
14. “N-acetyl cysteine” [Title/abstract]
15. #9 OR #10 OR #11 OR #12 OR #13 OR #14

16. OCD [Mesh Terms]
17. “obsessive compulsive disorder” [Title/abstract]
18. obsess* [Title/abstract]
19. compuls* [Title/abstract]
20. #16 OR #17 OR #18 OR #19

21. #8 AND #15 AND #20

Specific issues:

✓ [PT] stands for publication type and is one of the filters available in PubMed

✓ In CENTRAL, there is no need to use filters and keywords related to [T]

[MeSH] only can be used in PubMed, Central and ProQuest
